# PReS-FINAL-2079: Will cooling an arthritic joint cause damage? - an analysis of different cooling methods in patients with jia using thermography

**DOI:** 10.1186/1546-0096-11-S2-P91

**Published:** 2013-12-05

**Authors:** M König, F Kreuzpointner, M Hartmann, M Georgi, HJ Händel, A Schwirtz, JP Haas

**Affiliations:** 1 Deutsches Zentrum für Kinder- und Jugendrheumatologie Garmisch-Partenkirchen, Garmisch-Partenkirchen, Germany; 2 Department for Biomechanics in Sport, Technische Universität München, München, Germany

## Introduction

Physical therapy is one part of the multidisciplinary treatment in patients with juvenile idiopathic arthritis (JIA) (Spamer 2012). Usually joints with acute arthritis are treated with ice but apart from the analgetic effects the precise effects remain unknown. Local cooling may decrease the inflammatory reaction (Swenson, Sward et al. 1996) but there exist no systematic data about the optimal temperature and method of cooling. The temperature suitable to induce a benefit without causing harm has not been determined yet.

## Objectives

This study compares two different cooling methods according the surface temperature induced in an arthritic knee joint within a routine application.

## Methods

Nine JIA patients (age 13.1 ± 2.5 yrs) with unilateral gonarthritis had been included in a crossover design. Both methods were compared with two subsequent days of treatment. The healthy knee joint served as a control group. The cooling period was 12 minutes. Method A: cooling the joint with a classical kryo-pack with an initial temperature of about 5°C. Method B: was a cuff purged with water with constant temperature of 17°C allocated by a cooling system (Hilotherm Clinic, Hilotherm GmbH, Argenbuehl-Eisenharz, Germany). The observation period of the surface temperature around the patella was 20 minutes after cooling. Furthermore the linear fit of the rewarming curve (minimum temperature) after application has been calculated to examine the slope in terms of hyperemia - the steeper the fit, the higher the risk of hyperemia.

## Results

The initial mean temperature after cooling was 23.7 ± 3.2°C (method A) and 29.1 ± 2.1°C (method B) (p < .05). Twenty minutes after cooling skin temperature was 31.3 ± 3.1°C (A) and 31.9 ± 1.6°C (B) (p > .05). Compared to the control group (method A: 33.4 ± 1.8°C, method B: 33.1 ± 1.1°C) both methods produced significant lower values for the skin temperature after 12 minutes of cooling. The slope of both fits were statistical significant different (method A: 11.7 ± 5.2°, method B: 23.0 ± 5.0°; p < .01).

## Conclusion

Both methods reached a lower temperature after 12 minutes of cooling with respect to the control joint. They have proofed to have a similar effect according temperature and duration. But the main difference between both methods was that method B (29.1 ± 2.1°C) has a statistically significant higher temperature after a cooling period of 12 minutes than method A (23.7 ± 3.2°C) which might be more compatible. This could prevent from possible soft tissue damage.

The slope of the rewarming curve may serve as indicator for a faster rewarming effect which might cause hyperemia.

## Disclosure of interest

None declared.

